# Investigation of total skin helical tomotherapy using a 3D-printed total skin bolus

**DOI:** 10.1186/s12938-023-01118-7

**Published:** 2023-06-14

**Authors:** Haiyang Wang, Yifei Pi, Chunbo Liu, Xin Wang, Yuexin Guo, Lei Lu, Xi Pei, Xie George Xu

**Affiliations:** 1 Institute of Nuclear Medical Physics, University of Science and Technology of China, Hefei, China; 2 Department of Radiation Oncology, The First Affiliated Hospital of Zhengzhou University, Zhengzhou, China; 3 College of Information Science and Engineering, Henan University of Technology, Zhengzhou, China; 4 Anhui Wisdom Technology Co., Ltd, Hefei, Anhui China; 5 Department of Radiation Oncology, The First Affiliated Hospital of University of Science and Technology of China, Hefei, China

**Keywords:** 3D printing, Total skin bolus, Total skin helical tomotherapy, Mycosis fungoides

## Abstract

**Objective:**

To investigate the effectiveness of using a 3D-printed total skin bolus in total skin helical tomotherapy for the treatment of mycosis fungoides.

**Materials and methods:**

A 65-year-old female patient with a 3-year history of mycosis fungoides underwent treatment using an in-house desktop fused deposition modelling printer to create a total skin bolus made of a 5-mm-thick flexible material, which increased the skin dose through dose building. The patient's scan was segmented into upper and lower sections, with the division line placed 10 cm above the patella. The prescription was to deliver 24 Gy over 24 fractions, given 5 times per week. The plan parameters consisted of a field width of 5 cm, pitch of 0.287 and modulation factor of 3. The complete block was placed 4 cm away from the planned target region to reduce the area of the internal organs at risk, especially the bone marrow. Dose delivery accuracy was verified using point dose verification with a "Cheese" phantom (Gammex RMI, Middleton, WI), 3D plane dose verification with ArcCHECK (Model 1220, Sun Nuclear, Melbourne, FL), and multipoint film dose verification. Megavoltage computed tomography guidance was also utilized to ensure the accuracy of the setup and treatment.

**Results:**

A 5-mm-thick 3D-printed suit was used as a bolus to achieve a target volume coverage of 95% of the prescribed dose. The conformity index and homogeneity index of the lower segment were slightly better than those of the upper segment. As the distance from the skin increased, the dose to the bone marrow gradually decreased, and the dose to other organs at risk remained within clinical requirements. The point dose verification deviation was less than 1%, the 3D plane dose verification was greater than 90%, and the multipoint film dose verification was less than 3%, all of which confirmed the accuracy of the delivered dose. The total treatment time was approximately 1.5 h, which included 0.5 h of wearing the 3D-printed suit and 1 h with the beam on. Patients only experienced mild fatigue, nausea or vomiting, low-grade fever, and grade III bone marrow suppression.

**Conclusion:**

The use of a 3D-printed suit for total skin helical tomotherapy can result in a uniform dose distribution, short treatment time, simple implementation process, good clinical outcomes, and low toxicity. This study presents an alternative treatment approach that can potentially yield improved clinical outcomes for mycosis fungoides.

## Introduction

Mycosis fungoides (MF) is the most common type of cutaneous T-cell lymphoma, accounting for almost 50% of cases, with a mortality prognosis of up to 87% within 5 years [1]. Radiotherapy (RT) is an essential component of MF treatment, owing to the high radiosensitivity of MF [2]. Total skin electron irradiation (TSEI) is a traditional treatment method and is clinically regarded as one of the most effective methods for MF [3]. The dual-frame six-field irradiation technology, developed by the Stanford University School of Medicine, is currently widely used [4]. However, the large treatment area necessitates the patient standing and undergoing multifield irradiation with a rotating gantry, which can be a burden for the patient. Helical tomotherapy (HT) has several unique components that provide many advantages [5]. It is particularly effective for treating larger targets, up to 160 cm × 40 cm, in a single session. This makes it well-suited for the treatment of complex targets, such as total body multiple metastatic irradiation, craniospinal irradiation, total body irradiation, and total marrow irradiation, among others [6]. Total skin helical tomotherapy (TSHT) is superior to conventional TSEI, as it results in fewer setup errors, less fatigue, more comfort, and better dose distribution. Hsieh et al. [7] were the first to use a 3 mm diving suit as a bolus to perform TSHT. The diving suit covered the whole body to increase the superficial dose, and the central core complete block (CCCB) technique was used to reduce the internal organ dose. The tumour regressed continuously without further nodular plaques. This method was able to treat 95% of the target volume at the prescribed dose (30 Gy). Haraldsson et al. [8] achieved treatment of 95% of the target volume with more than 95% of the prescribed dose (12 Gy). However, due to the dose-building effect of photons, it is challenging to administer doses to the skin surface. The use of a low-density bolus, such as a diving suit, commonly results in underdosing relative to the prescription, and a high-density bolus is required to reach the prescribed dose. Electron therapy, which does not require a bolus, is an alternative method for increasing the skin dose. Deveau et al. [9] used 3D printing technology to investigate the effect of changing the bolus on increasing skin dose in TSHT. Specifically, they created a total skin bolus for a dog using 3D printing technology. Additionally, Baltz et al. [10] used 3D printing technology to make a total scalp bolus to achieve total scalp irradiation. Thermoplastic urethane (TPU) suits made with 3D printing technology can easily be used to increase the skin dose by varying the density and thickness. The printer uses fused deposition modelling (FDM) technology with a limited printing volume range (40 × 40 × 30 cm), and multiple segments are spliced to form the total skin bolus for the patient. To date, there are no reports on the use of 3D printing technology to make a total skin bolus for TSHT in MF treatment. The purpose of this study was to explore the use of 3D printing technology to create a total skin TPU suit for use during TSHT as an alternative treatment method with potentially better clinical outcomes for MF treatment.

## Results

### Dosimetric parameters of target volumes

**Fig. 1:**
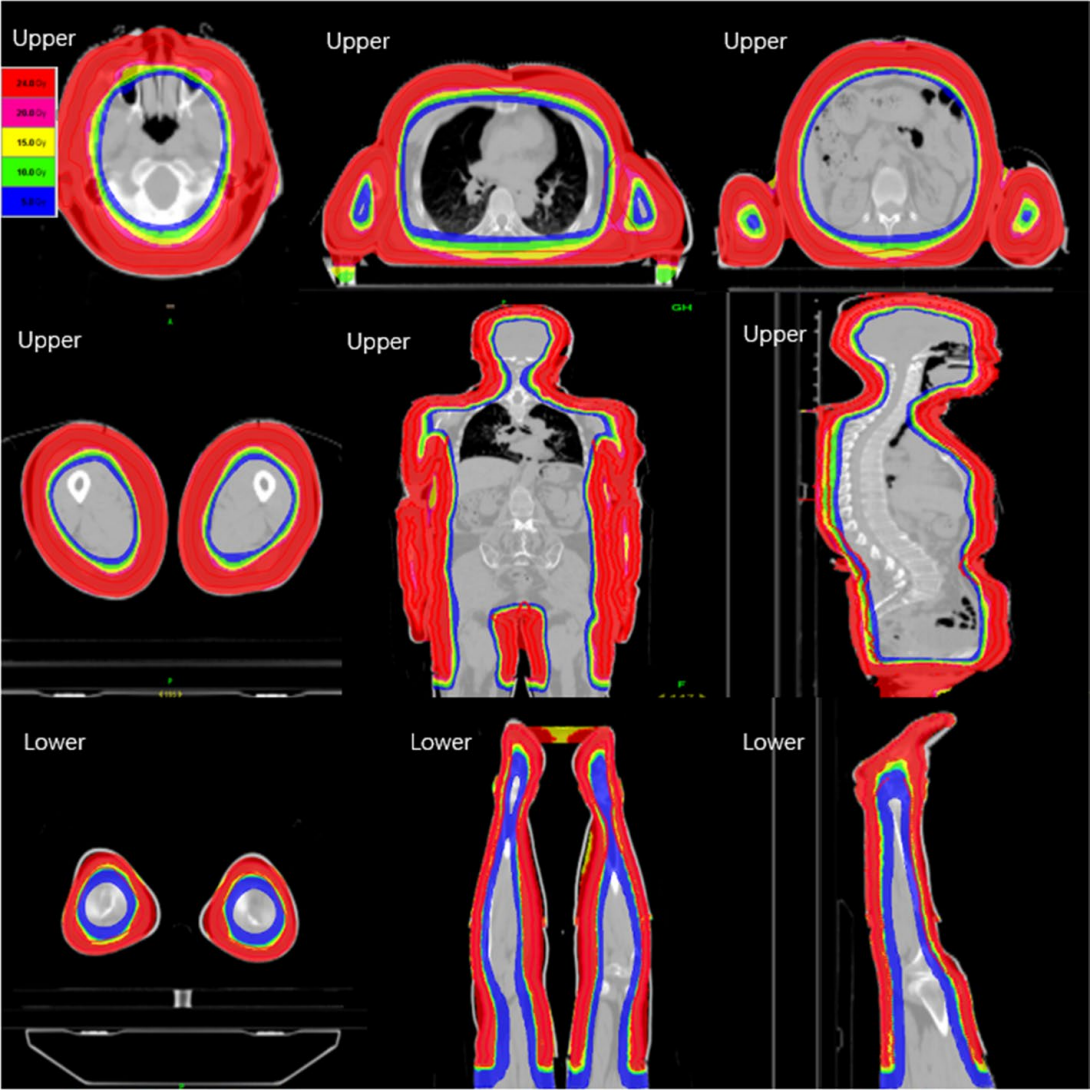
Dose distribution of the upper and lower segments in the transverse, coronal and sagittal planes

The upper and lower segments were designed separately, with the upper segment consisting of four parts: the head and neck, the thorax and abdomen, the left arm and the right arm. The quality assessment results for each part are presented in Table 1. The lower segment achieved closer adherence to the prescription dose than the upper segment, with the conformity index (CI) and homogeneity index (HI) of the lower segment slightly superior to those of the upper segment in the transverse, coronal, and sagittal plane dose distributions (Fig. 1).

**Table 1:** PTV_mean_, HI, and CI of the upper segment and lower segment, including four subtargets (Gy, $\overline{x }\pm s$)

Site/result	PTV_*mean*_(Gy)	HI	CI
Lower	24.92 ± 1.65	1.10	0.89
Upper	25.48 ± 1.98	1.14	0.75
Upper_Head&Neck	25.45 ± 1.23	1.12	N/A^a^
Upper_Thorax&Abdomen	25.82 ± 1.31	1.13	N/A^a^
Upper_Arm_L	25.33 ± 2.03	1.38	N/A^a^
Upper_Arm_R	25.43 ± 2.10	1.34	N/A^a^

^a^There is no corresponding V_ref_ for the subtargets, so there is no CI value

### Dosimetric parameters of auxiliary structures

The mean doses of bone marrow for bone_leg, bone_H&N, bone_pelvic, bone_spinal, bone_rib, bone_arm, and bone_femur were 5.99 ± 4.68 Gy, 18.03 ± 9.58 Gy, 6.84 ± 6.41 Gy, 3.46 ± 1.46 Gy, 9.52 ± 8.77 Gy, 21.16 ± 5.68 Gy, and 3.57 ± 0.43 Gy, respectively (Table 2). The mean doses to parallel organs at risk (OAR), such as the left and right parotid, left and right lungs, left and right kidneys, left and right breast, heart, liver, stomach, oral cavity, and pituitary were 25.07 ± 5.81 Gy and 25.44 ± 5.75 Gy, 5.36 ± 1.96 Gy and 5.26 ± 2.18 Gy, 6.94 ± 3.44 Gy and 7.41 ± 3.68 Gy, 25.15 ± 5.08 Gy and 25.92 ± 4.20 Gy, 5.66 ± 3.26 Gy, 6.16 ± 5.65 Gy, 3.46 ± 0.55 Gy, 11.42 ± 8.92 Gy, and 3.51 ± 0.56 Gy, respectively (Table 2). The maximum doses to serial organs at risk, such as the left and right lens PRV03, left and right optic nerve, optic chiasm, brainstem, small bowel, and spinal cord were 8.67 Gy and 8.68 Gy, 22.66 Gy and 20.32 Gy, 3.02 Gy, 2.73 Gy, 2.73 Gy, and 6.21 Gy, respectively (Table 2). Overall, the total body bone marrow dose increased gradually as the distance from the skin decreased, but all OAR doses were within the clinically acceptable tolerance range.

**Table 2:** OAR dose statistics (Gy, $\overline{x }\pm s$)

Dose	D_mean_	D_max_	Dose	D_mean_	D_max_
OARs			OARs		
Bone_Leg	5.99 ± 4.68	29.82	Heart	5.66 ± 3.26	14.81
Bone_H&N	18.03 ± 9.58	30.22	Liver	6.16 ± 5.65	29.97
Bone_Pelvis	6.84 ± 6.41	30.09	Stomach	3.46 ± 0.55	9.48
Bone_Spinal	3.46 ± 1.46	16.41	Cavity_Oral	11.42 ± 8.92	30.18
Bone_Rib	9.52 ± 8.77	30.16	Pituitary	3.51 ± 0.56	4.63
Bone_Arm	21.16 ± 5.68	30.45	Lens_L_PRV03	6.07 ± 1.10	8.67
Bone_Femur	3.57 ± 0.43	6.27	Lens_R_PRV03	6.18 ± 1.25	8.68
Parotid_L	25.07 ± 5.81	29.94	OpticNrv_L	10.06 ± 6.36	22.66
Parotid_R	25.44 ± 5.75	29.98	OpticNrv_R	8.38 ± 4.95	20.32
Lung_L	5.36 ± 1.96	22.53	OpticChiasm	2.40 ± 0.23	3.02
Lung_R	5.26 ± 2.18	14.02	Brainstem	2.13 ± 0.13	2.73
Kidney_L	6.94 ± 3.44	15.48	Bowel_Small	7.03 ± 6.35	30.57
Kidney_R	7.41 ± 3.68	16.78	SpinalCord	3.69 ± 0.56	6.21
Breast_L	25.15 ± 5.08	30.27	External_Up	15.25 ± 9.09	30.96
Breast_R	25.92 ± 4.20	30.28	External_Down	14.26 ± 10.42	28.51

### Point dose verification

The Tomotherapy “Cheese” phantom and an A1SL ionization chamber from Standard Imaging (Middleton, WI) were utilized for point dose verification. The percent dose difference was calculated using the formula: difference = (Dm-Dc)/Dc*100%, where Dm and Dc represent the measured dose and calculated dose, respectively. Smaller percent dose differences closer to 0 indicate better accuracy, and all percent dose differences were less than 3% [11, 12]. The measurement results (Table 3) revealed that the differences were all within 1%, meeting the clinical requirements.

**Table 3:** Point dose measurements with the “Cheese” phantom, calculation results and corresponding difference

Site/result	Calculation (Gy)	Measurement (Gy)	Difference (%)
Upper_Head	1.076	1.081	0.465%
Upper_Thorax	1.109	1.116	0.631%
Upper_Abdomen	1.121	1.129	0.714%
Lower	1.089	1.095	0.551%

### 3D Plane dose verification

**Fig. 2:**
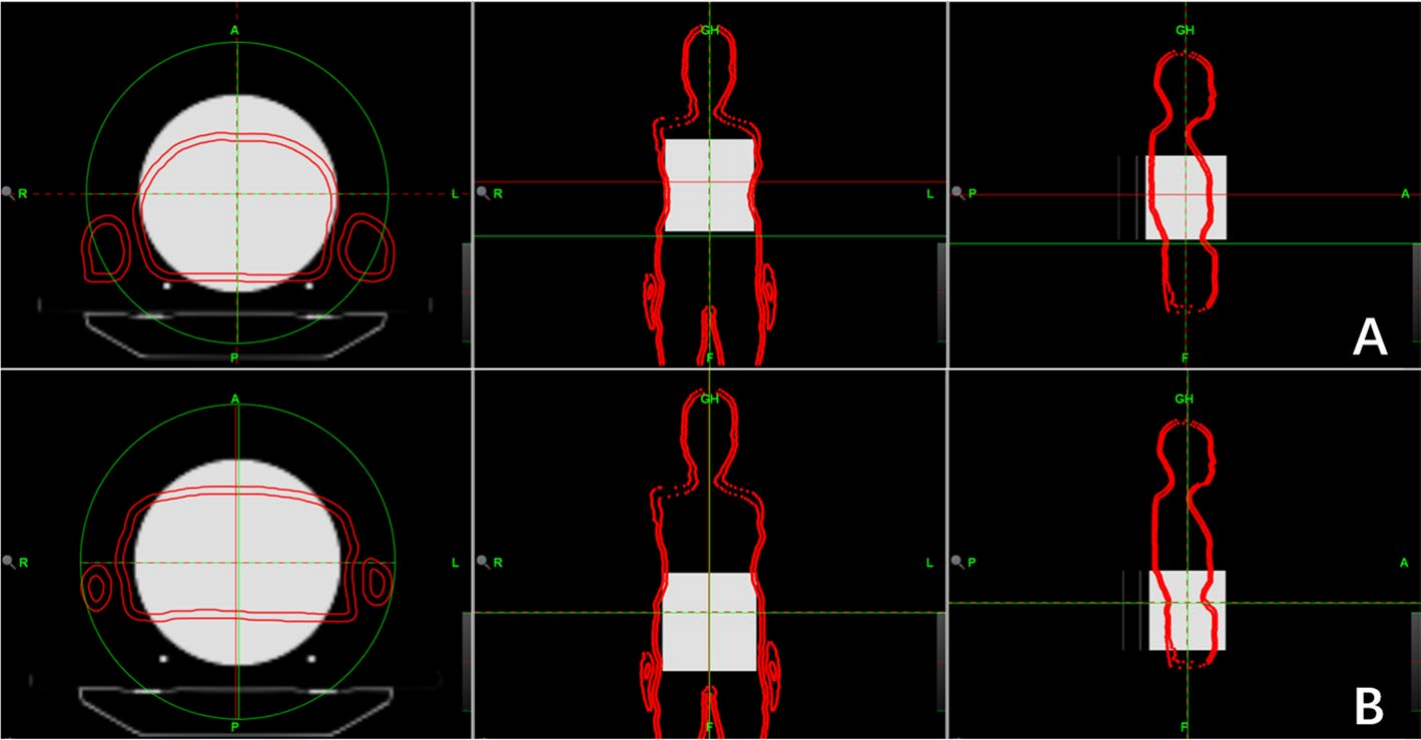
Dose map of ArcCHECK in the thorax and abdomen regions

ArcCHECK was utilized for 3D plane dose verification of the upper and lower segments, head, thorax (Fig. 2A), and abdomen (Fig. 2B). The gamma passing rates were calculated using three different criteria: TG119 [11] with a 3%/3 mm, 10% threshold, and TG218 [12] with a 3%/2 mm, 10% threshold. The passing rates were all at least 95% and 90%, respectively. The verification results (Table 4) indicated that the passing rate was above 90%, meeting the clinical requirements.

**Table 4:** ArcCHECK 3D plane dose verification results

Site/result	Passing rate (3%/3 mm)	Passing rate (3%/2 mm)
Upper_Head	96.3	91.8
Upper_Thorax	95.8	91.3
Upper_Abdomen	95.1	90.2
Lower	96.9	91.9

### Multipoint film dose verification

**Fig. 3:**
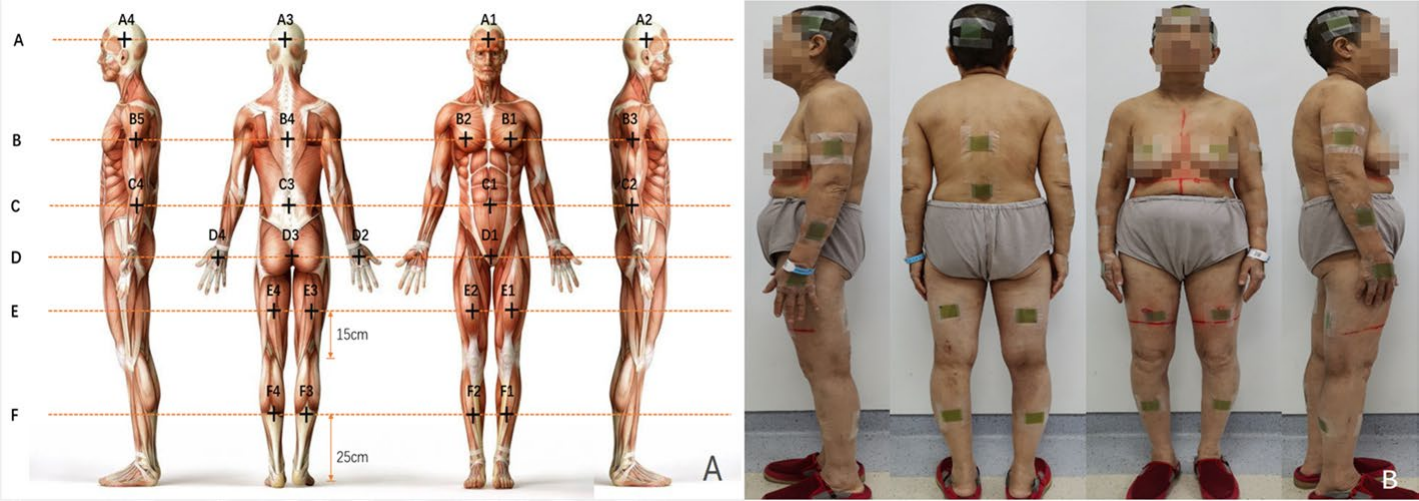
Schematic diagram of simulated and measurement points for film verification

The Gafchromic EBT3 film was used to obtain multiple point doses, which is considered a gold standard for dose measurements in TomoTherapy [13, 14]. The whole film was divided into smaller pieces, each with a size of 4 cm × 5 cm, and placed in the corresponding simulated position (Fig. 3A) to obtain the actual point dose (Fig. 3B). During MVCT and treatment, the patient wore a 3D-printed TPU suit and carried the split film. The films were exposed during image acquisition in TomoTherapy, and the MVCT only increased by 1.0 cGy–2.85 cGy [15], which is approximately 1% – 2% relative to the prescribed dose of 120 cGy. The measurement results using the film (Table 5) showed that most of the values were within 3%, while the regional deviation of excessive motion range (such as both nipples, navel, and pubic symphysis) was within 5%, meeting the clinical requirements.

**Table 5:** Multipoint dose measurement results with film, calculation results and corresponding difference

Site/result	Calculation (Gy)	Measurement (Gy)	Difference (%)
A1	109.11	110.05	0.86
A2	111.05	112.43	1.24
A3	106.55	109.71	2.97
A4	113.95	115.39	1.26
B1	111.08	115.18	3.69
B2	112.85	116.82	3.52
B3	108.65	111.99	3.07
B4	112.50	108.91	− 3.19
B5	109.85	112.80	2.69
C1	110.25	113.60	3.04
C2	114.15	117.99	3.36
C3	104.25	101.11	− 3.01
C4	111.45	114.80	3.01
D1	108.15	110.86	2.51
D2	102.10	106.93	4.73
D3	113.10	110.80	− 2.03
D4	103.50	108.45	4.78
E1	107.45	109.56	1.96
E2	108.53	110.06	1.41
E3	111.10	113.52	2.18
E4	112.75	115.25	2.22
F1	109.35	110.99	1.50
F2	108.25	110.24	1.84
F3	110.35	113.99	3.30
F4	112.25	115.24	2.66

### TSHT treatment

**Fig. 4:**
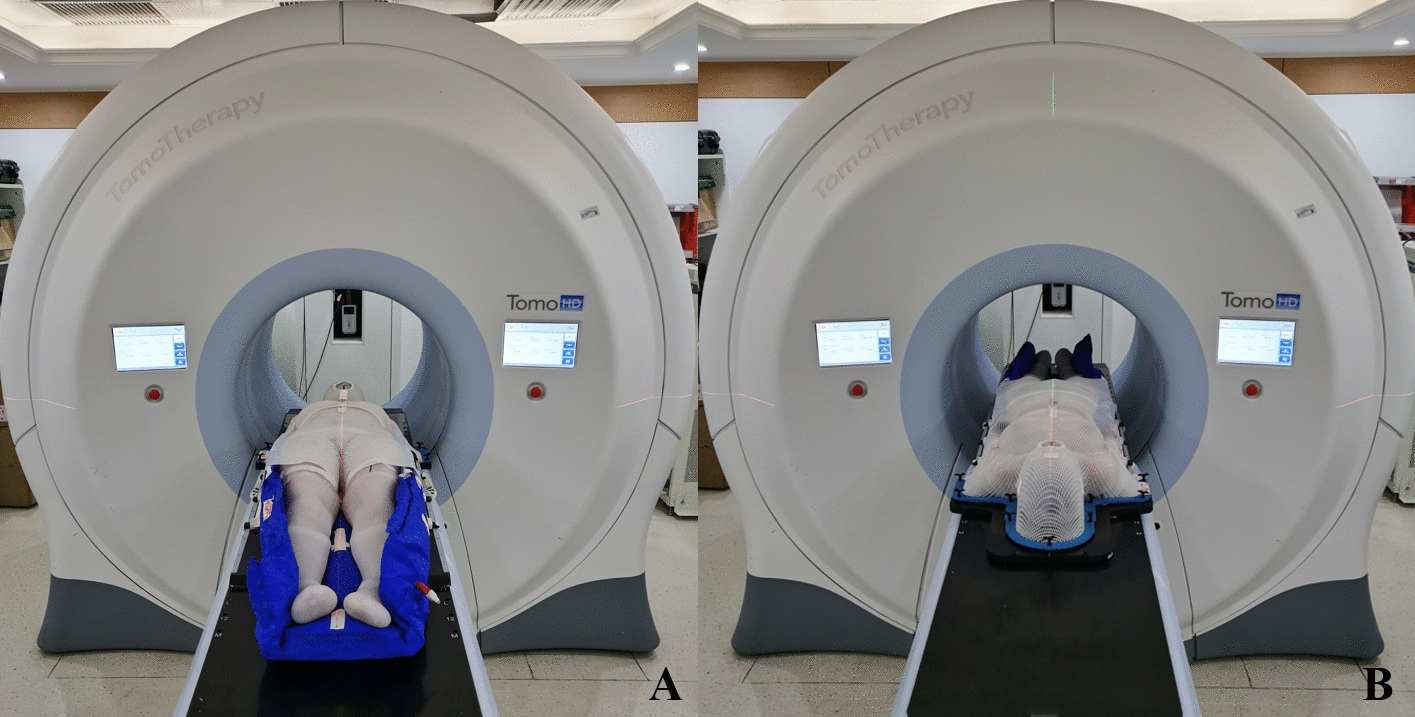
The patient was treated first in a head-first supine position for the upper segment (**A**) and then in a feet-first supine position for the lower segment (**B**)

MVCT was performed before each treatment to ensure setup accuracy. The maximum setup tolerance was less than 5 mm in all three dimensions, and the maximum axial rotation tolerance was less than 1 degree. For the upper segment, which was relatively long, we used the average correction of the third cervical vertebra and the fifth lumbar vertebra for both setup and treatment. If the average deviation was greater than 5 mm, the patient was repositioned. For the lower segment, the area near the patella was scanned, and if the deviation was greater than 5 mm, the patient was repositioned. If the deviation was less than 5 mm, the position was corrected directly. The patient was treated first in a head-first supine position for the upper segment (Fig. 4A) and then in a feet-first supine position for the lower segment (Fig. 4B). The beam-on time was 1519.3 s for the upper segment, 637.7 s for the lower segment, and a total of 2157 s for both segments.

### Radiation toxicity

To ensure the safety of the elderly patient, haematology tests, including complete biochemical tests, erythrocyte sedimentation rate, coagulation, and routine imaging (such as B-ultrasound, CT, and PET-CT), were performed during the treatment period. However, after 12 fractions, radiotherapy was discontinued due to the patient's symptoms of fatigue, nausea, vomiting, and low-grade fever. The patient developed grade III bone marrow suppression, with a platelet count drop to 50 × 10^9^/L and a white blood cell count drop to 2.0 × 10^9^/L. Considering the patient's older age and treatment safety, the physician decided to terminate the treatment. The patient was administered recombinant human granulocyte stimulating factor and recombinant human thrombopoietin, and after 1 week of continuous recovery, the patient's haematological values returned to the clinically normal range, and their adverse reaction was improved.

## Discussion

Based on our previous experience with TSHT, where six patients were treated using diving suits, we monitored this patient throughout the entire TSHT treatment process [16]. To overcome the issue of insufficient skin dose deposition, we utilized a customized 3D-printed TPU suit as a bolus. The suit allowed for uniform dose distribution, a good fit, a simple implementation process, effective treatment outcomes, and minimal toxic side effects. Overall, this approach offers an alternative treatment method with superior clinical outcomes for the treatment of MF.

In this study, a 5 mm TPU suit created by 3D printing was utilized as a bolus to enhance the skin dose. Previous research by Hsieh et al. [7] employed a 3 mm diving suit as a bolus to achieve 90% of the prescribed dose to the target area. Schaff et al. [17] studied two MF patients and demonstrated that TSHT can replace traditional TSEI using a 3 mm diving suit as a bolus. Film verification showed that a diving suit can considerably increase the skin dose. In another study, Deveau et al. [8] treated a dog with cutaneous epitheliotropic lymphoma, utilizing 3D printing technology to create a 10-mm-thick 3D mould scaffold with a density of 1.09 g/cm^−3^, resulting in 92% of the target reaching the prescribed dose. Baltz et al. [10] employed 3D printing technology to manufacture a total scalp bolus for total scalp irradiation treatment. To our knowledge, there are no published studies on the application of 3D printing technology to fabricate a total skin bolus for MF treatment using TSHT.

Compared to the lower segment, the upper segment had a slightly worse dose distribution, with consistent CI and HI. This was mainly due to the lateral width of the patient's left and right arms being greater than 40 cm, resulting in blind areas at certain angles that could not be irradiated. Sarfehnia et al. [18] also observed over or underdose in the left and right arms during the TSHT of a child. Further research is needed to develop a method to manage the doses in the left and right arms.

The highest radiation toxicity is observed in bone marrow suppression, as reported in previous literature. Schaff et al. [17] observed grade IV bone marrow suppression with a mean dose of 1.66 Gy when TSHT was used at 12 Gy with 8 fractions. Hsieh et al. [7] also reported similar bone marrow suppression rates at a higher prescribed dose of 30 Gy. One possible explanation for this is that the TPS may not accurately simulate the actual bone marrow dose, or a plan parameter with a mean bone marrow dose less than 2 Gy is not stringent enough for TSHT. To minimize toxicity, we outlined the total bone marrow areas individually, including bone_leg, bone_H&N, bone_pelvic, bone_spinal, bone_rib, bone_arm, and bone_femur, and included strict dose limitations for bone marrow in the treatment plan. The bone marrow dose showed significant variation closer to the skin. Bone_H&N and bone_femur, which are farther from the skin, received slightly lower doses compared to bone_pelvis and bone_spinal. In situations where the choice needs to be made between reducing the bone marrow dose and losing part of the target or increasing the bone marrow dose to ensure the target receives the prescribed dose, the physician should make a decision based on the patient's individual situation. Although using a 3D-printed TPU suit as a bolus increased skin dose deposition, in this case, the patient was thin, and part of the bone marrow was close to the skin, making it difficult to reduce the dose sufficiently. As a result, the patient experienced grade III bone marrow suppression after the 12th fraction, and the treatment had to be terminated.

MF is a highly radiosensitive disease, and radiotherapy is a recommended treatment method [19]. The prescribed dose for MF treatment can vary widely depending on the treatment goal, with 15–20 Gy being sufficient for palliative treatment. However, recent studies have shown that the complete remission rate for 10–20 Gy is only 55%, while a dose of 30 Gy or more can result in a complete remission rate of 94%. Nevertheless, a single course of treatment should generally not exceed 36 Gy to avoid severe acute phase responses [2]. The European Organization for Research and Treatment of Cancer (EORTC) recommends a prescription dose of 30–36 Gy for 6–10 weeks, with at least 26 Gy administered in a cone-shaped region of skin at a depth of 4 mm along the central axis for cutaneous lymphoma [20]. However, the low-dose model has been gaining popularity in recent years due to its shorter treatment time and lower toxicity effects. In this study, a prescribed dose of 24 Gy in 24 fractions was selected. The choice of the prescribed dose and number of fractions should be made based on the patient's actual situation. Different research institutes have used varying prescription doses and fractions. Hsieh et al. [7] used 30 Gy in 40 fractions; Schaff et al. [17] used 12 Gy in 8 fractions; and Haraldsson et al. [8] used 32 Gy in 24 fractions. Therefore, institutes need to choose an appropriate prescription dose and number of fractions according to the actual situation of the patient [21].

The measured dose was found to be consistent with the calculated dose, which is in agreement with the findings of Akbas et al. [22]. However, deviations exceeding 3% were observed for D2 and D4 due to bolus fit and involuntary movements, although they were less than 5%. Deviations for B4, C3, and D3 were lower due to better bolus fit and slight involuntary movement. For B3, B5, C2, C4, D2, and D4 on the left and right arms, larger degrees of freedom and poor repeatability resulted in slightly larger regional deviations from the shoulder to the palm than those of the total body, but all deviations were within 3%. Segmented treatment could have contributed to the deviation, as the measured films may have received scattered radiation during treatment of other segments. Inaccurate calculation of the surface dose by the planning system could also contribute to dose deviations [23]. Overall, most of the deviations were within 3%, and even the regional deviations resulting from excessive motion were within 5%, thus ensuring the accuracy of the delivered dose.

The upper segment had a beam-on time of 1519.3 s, the lower segment had a beam-on time of 637.7 s, and the total beam-on time was approximately 2157 s (25.3 min). Although the customized TPU suit was not as convenient as a conventional diving suit and required an additional half hour to wear in the treatment room, the preparation time, setup time, and MVCT image guidance time added up to one and a half hours. Compared with the two and a half hours required for TSEI, the time was shortened by nearly half [24], which significantly improved treatment efficiency. The patient was more comfortable and could maintain position repeatability better in the supine position than in the traditional standing position. In addition, the intensity-modulated treatment plan greatly improved HI and CI, ensuring the accuracy and safety of the treatment. Please review the above sentences.

The difference between the measured dose and calculated dose for TSEI can be as high as 40%, and in some regions, such as the perineum and eyelids, it can be as high as 90% [25]. Additionally, TSEI may provide an insufficient dose for skin tumors with a depth greater than 4 mm. Compared to TSEI, HT has advantages for larger targets because it provides a uniform dose, precise dose depth control, and low organ toxicity [23]. Therefore, TSHT can be used instead of TSEI. In this case, the upper and lower targets received 95% of the prescribed dose, and the maximum dose was 115%, resulting in a smaller dose deviation than TSEI. Furthermore, the complete block was placed 4 cm away from the PTV, significantly reducing the internal OAR dose and lowering the incidence of toxicity.

Hsieh et al. [7] successfully treated 95% of the target volume at the prescribed dose (30 Gy). Haraldsson et al. [8] delivered more than 95% of the prescribed dose (12 Gy) to 95% of the target volume. Sarfehnia et al. [18] treated 95% of the target volume at the prescribed dose (14 Gy). Lin et al. [26] delivered more than 95% of the prescribed dose (36 Gy) to 95% of the target volume. In this study, we were able to achieve a 95% treatment of the target volume at the prescribed dose (24 Gy) with ease, thanks to the use of 3D printing technology for the fabrication of total skin boluses. In summary, 3D printing technology offers a more efficient way of delivering prescription doses than using diving suits as boluses.

As the TPU material is soft and elastic, margins were reserved at the junctions between parts to allow the bolus to be worn conveniently. The margins can be trimmed on demand, which means that the 3D-printed suit can be cut like a zipper opening to put it on the patient and then closed like a zipper after it is on. However, this can cause air gaps to occur between the TPU and the skin, especially in the armpit and thigh area. The gaps between the TPU and the skin may become apparent due to the breathing movement of the chest. The next step will be to zip up the openings of the TPU coat to enable the TPU to fit the skin more closely while still being easily removable, thereby reducing the gap.

Hsieh et al. [7] used a prescription dose of 30 Gy in 40 fractions; Schaff et al. [17] used 12 Gy in 8 fractions; Haraldsson et al. [8] used 32 Gy in 24 fractions; Sarfehnia et al. [18] used 14 Gy in 7 fractions, and in this study, we used a prescription dose of 24 Gy in 20 fractions. It should be noted that each research unit used different prescription doses, and therefore direct comparison of OAR doses between studies may not be appropriate.

The mean surface dose of the lesion measured by Hsieh et al. [7] was approximately 84.0 cGy, which corresponded to approximately 120% of the prescription dose. Haraldsson et al. [8] reported a mean surface dose difference of 5.3% between the measured dose and the TPS calculation. Sarfehnia et al. [18] found that the difference between the measured and calculated doses was within 5% in the regions of interest. In this study, the difference between the film measurements and TPS calculations was also within 5%, consistent with the findings of the previous studies.

Due to the dose-building effect of photons, they are not conducive to skin dose deposition or the purpose of improving the skin dose by increasing the bolus, while electrons do not require a bolus due to the lack of a dose-building effect. This means that electrons easily achieve the necessary skin dose due to their short effective range and that the energy is mainly deposited on the skin surface; thus, there is no need to increase the skin dose by using a bolus. To date, there have been no reports on TSHT of MF using a total skin bolus fabricated by 3D printing technology. The purpose of this work was to use 3D printing technology to create a TPU-based suit for TSHT to provide another treatment method with better clinical effects for the treatment of MF.

The patient carried the split film and wore the 3D-printed TPU suit during both MVCT and treatment. The films were exposed during image acquisition in tomotherapy, and the additional dose delivered by MVCT was negligible, ranging from 1.0 to 2.85 cGy [15], which is approximately 1–2% relative to the prescribed dose of 120 cGy. The film measurements (Table 5) indicated that most of the deviations were within 3%, and the regional differences in excessive motion range were within 5%, such as in the areas of both nipples, navel, and pubic symphysis, which met the clinical requirements.

The decision to use a 5-mm-thick bolus was based on previously published research results, as well as practical considerations such as patient comfort and fit. Previous experience with using a diving suit as a bolus was also taken into account. There is no strict requirement for the bolus density to be greater than that of water; it is only necessary for the density to be close to that of water.

The manuscript comprises of five tables and four figures. Table 1 displays the quality assessment results of the head and neck, thorax and abdomen, left arm and right arm. Table 2 shows the mean doses of bone_leg, bone_H&N, bone_pelvic, bone_spinal, bone_rib, bone_arm, and bone_femur for bone marrow; the mean doses of the left and right parotid, left and right lungs, left and right kidneys, left and right breast, heart, liver, stomach, oral cavity, and pituitary for the parallel OARs; and the maximum dose of the left and right lens PRV03, left and right optic nerve, optic chiasm, brainstem, small bowel, and spinal cord for serial OARs. Table 3 displays the results of point dose measurements, calculation results, and corresponding differences for the “Cheese” phantom. Table 4 shows the results of ArcCHECK 3D plane dose verification for the upper and lower segments. Table 5 displays the results of multipoint film dose verification, calculation results, and corresponding difference. Figure 1 illustrates the dose distribution of the upper and lower segments in the transverse, coronal, and sagittal planes. Figure 2 shows the ArcCHECK used for the 3D plane dose verification of the upper and lower segments. Figure 3 depicts a schematic diagram of the simulated and measurement points for film verification. Finally, Fig. 4 illustrates the patient in a head-first supine position for the upper segment and in a feet-first supine position for the lower segment.

## Conclusion

The combination of a 3D-printed suit and helical tomotherapy provides a promising approach for total skin irradiation in patients. TSHT with a 3D-printed suit can deliver a uniform dose distribution with a short treatment time, simple implementation process, low toxicity, and excellent clinical outcomes. This study presents a novel treatment option with superior clinical efficacy for mycosis fungoides patients.

## Materials and methods

### Bolus

Before treatment, a CT scan of the patient's entire body was performed, and the resulting data was imported into the Mimics17 software in the DICOM format. The patient's external contour was then reconstructed and output in STL format for 3D printing. To create a more flexible and simulation-like bolus, a specific type of elastic printing material was chosen: thermoplastic polyurethane (TPU) filaments with a diameter of 1.75 mm, with a thickness of 5 mm and a density of 1.10–1.14 g/cm^3^. The design of the total body bolus was based on clothing structure, and the bolus was fabricated in parts based on the printer volume. Because the 3D printer, which utilized fused deposition modeling (FDM) technology, had a limited range, multiple segments were joined together to form a complete total skin bolus for the patient. Two printers were used to print the bolus, and the entire printing process took 5 days. As shown in Fig. 4A, the patient wore the 5 mm TPU bolus, which was custom-fitted to the patient's external shape to facilitate easier donning and doffing.

### Preparatory stage

The patient, a 65-year-old female with a 3-year history of MF, was admitted to the Department of Radiation Oncology of the First Affiliated Hospital of Zhengzhou University in June 2021 to undergo pre-TSHT. The clinical diagnosis indicated that the stage was T_4_N_0–2_M_0_B_0_.

**Fig. 5:**
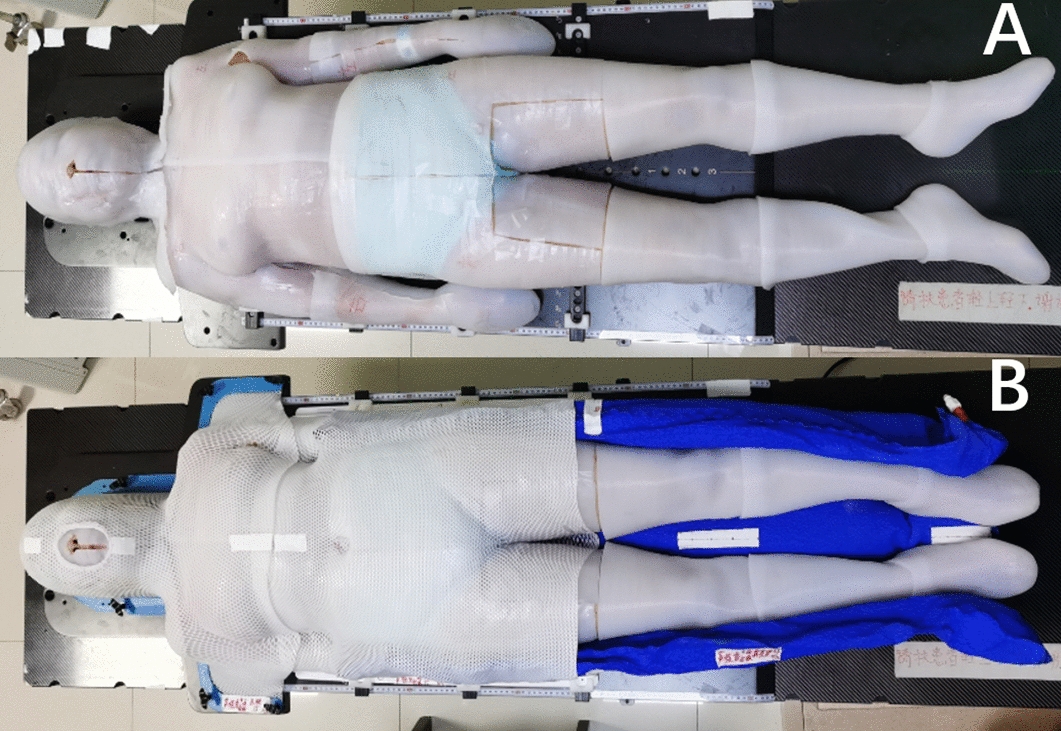
The patient wearing the TPU suit in the immobilization position

The patient was immobilized in a supine position using the 5 mm TPU suit (Fig. 5B). Thermoplastic masks were used to immobilize the head, neck, thorax, and abdomen, while the lower limbs were immobilized in a vacuum cushion.

**Fig. 6:**
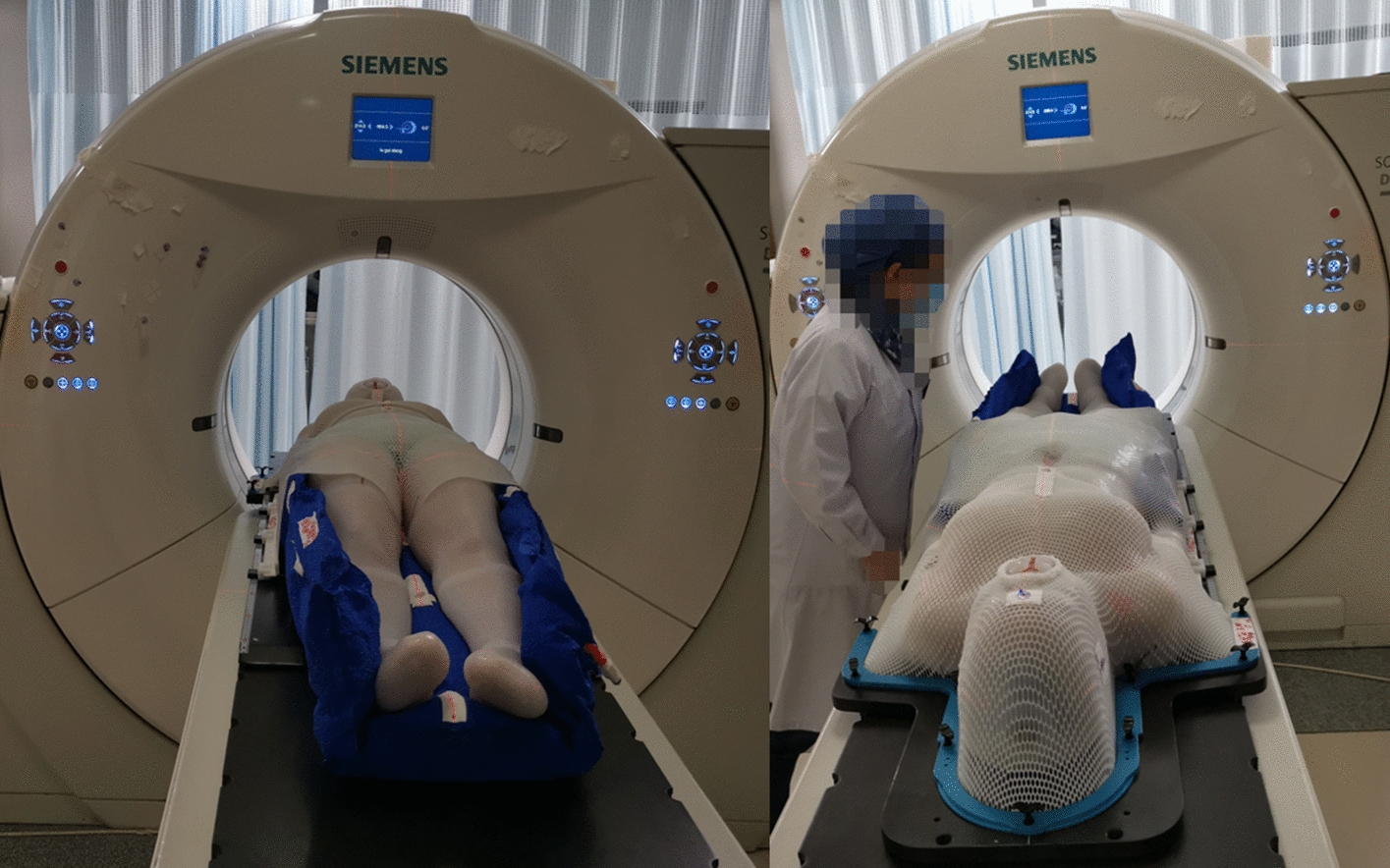
Patient scans of the upper and lower segments

The CT scans were conducted using a SOMATOM Definition AS40 scanner from Siemens, with a reconstruction slice thickness of 5 mm. The patient's body was divided into upper and lower segments, with a lead segment line placed approximately 10 cm above the patella, while the upper and lower marks were positioned near the patient's belly button and patella, respectively. The upper segments were scanned from the skull to 10 cm below the boundary, whereas the lower segment was scanned from the toes to 10 cm above the boundary (Fig. 6).

### Delineation of target volumes and organs at risk

The two sets of CT images were transferred to the physician’s workstation (Eclipse 13.5; Varian, Palo Alto, CA, USA) for delineation. The target volumes were delineated by radiation oncologists based on the planning CT images according to the ICRU83 [27] and ICRU91 [28]. The clinical target volume (CTV) was defined as the volume up to a depth of 5 mm below the skin surface [7]. To create the planning target volume (PTV), the CTV was expanded by 5 mm and then retracted by 3 mm in the outside region. OARs were delineated based on the ICRU83 report [27], including the total bone marrow (bone_leg, bone_H&N, bone_pelvic, bone_spinal, bone_rib and bone_arm, etc.), parotid, lung, kidney, breast, heart, and liver. The junction between the upper and lower segments of total body irradiation (TBI) has been previously studied, and it was found that the dose in the overlap region was mostly homogeneous when the distance was equal to the field width [29].

### Plan designs

The planning CT images and contoured structures of the patient were imported to the treatment planning system (Version 5.1.6; Accuray, Sunnyvale, CA, USA). The prescribed dose was 24 Gy delivered in 20 fractions, 5 times per week, following the RTOG guidelines. An auxiliary structure called the remaining center volume was set to complete mode at a distance of 4 cm from the PTV [16] for plan optimization to achieve dose control. Two plans were designed for the upper and lower targets, with a field width of 5 cm, modulation of 3, pitch of 0.287, and dose grid of 0.195 cm × 0.195 cm.

### Evaluation of plan quality

The patient parameters evaluated included the mean dose, heterogeneity index (HI), and conformity index (CI) of the target volume. The prescribed dose was delivered to at least 95% of the target volume. The HI was calculated using the following formula:

1 $${\text{HI}} = \frac{{{\text{D5\% - D95\% }}}}{{{\text{D95\% }}}}$$

where D_5%_ is the dose received by 5% of the PTV and D_95%_ is the dose received by 95% of the PTV. An HI value of 0 represents the heterogeneity dose distribution of the target volume. The CI was obtained using the following Paddick equation [30]:

2 $${\text{CI}} = \frac{{{\text{V}}_{{{\text{T,ref}}}} }}{{V_{T} }} \times \frac{{{\text{V}}_{{{\text{T,ref}}}} }}{{V_{{ref}} }}$$

where V_T,ref_ is the target volume covered by the prescription isodose (cm^3^), V_ref_ is the volume covered by the prescription isodose (cm^3^), and V_T_ is the target volume (cm^3^). The closer the CI value is to 1, the better the dose conformity of the target volume. Strict requirements were implemented for the dose of OARs; the maximum dose of the lens plan risk volume (PRV), the mean dose to the lung, the mean dose to the left and right kidneys, and the mean dose to the liver were less than 9 Gy, 8 Gy, 7 Gy, and 8 Gy [31], respectively. Bone marrow is very sensitive to radiation and is considered the most important OAR during treatment. It has been confirmed that the total dose of bone marrow irradiation is related to blood toxicity, especially the side effects of bone marrow suppression in the skull; the mean dose to the ribs and sternum was minimized under the premise of safety [17].

### Dose verification

Three dose verification techniques were performed, including point dose verification using a "Cheese" phantom, 3D plane dose verification using ArcCHECK, and total body multipoint film verification using US Gafchromic EBT3 film. The gamma analysis criteria were based on TG119 [11] and TG218 [12]. The gamma passing rates for the 3%/3 mm and 3%/2 mm criteria were above 95% and 90%, respectively, and the point dose deviation was less than 3%.

### TSHT treatment

Image guidance radiotherapy (IGRT) was performed for every treatment, and MVCT was performed for setup verification three times. The image acquisition length for the upper and lower segments in IGRT was approximately 15 cm. Normal image reconstruction was used, with a default reconstruction slice thickness of 2 mm. To account for the relatively long upper segment, the neck and waist were averaged for setup and treatment correction. The patella was used for setup and treatment correction of the lower segment.

### Clinical observation

To ensure the safety of the elderly patient, regular haematology tests were performed during the treatment, including routine blood tests every 2 days, complete biochemical tests, erythrocyte sedimentation rate, and weekly coagulation tests. Routine imaging, including B-ultrasound, CT, or PET-CT, was also performed. After 10 fractions, the patient's blood indicators started to decline, and after 12 fractions, radiotherapy was stopped due to symptoms of fatigue, nausea, vomiting, low-grade fever, and grade III bone marrow suppression.

## Data Availability

Written informed consent was obtained from the individual for the publication of any potentially identifiable images or data included in this article. The data are not publicly available due to privacy concerns and ethical restrictions.
